# Runx2-I Isoform Contributes to Fetal Bone Formation Even in the Absence of Specific N-Terminal Amino Acids

**DOI:** 10.1371/journal.pone.0108294

**Published:** 2014-09-22

**Authors:** Hideaki Okura, Shintaro Sato, Sari Kishikawa, Satoshi Kaneto, Tomoki Nakashima, Nobuaki Yoshida, Hiroshi Takayanagi, Hiroshi Kiyono

**Affiliations:** 1 Division of Mucosal Immunology, Department of Microbiology and Immunology, The Institute of Medical Science, The University of Tokyo, Tokyo, Japan; 2 Department of Medical Genome Science, Graduate School of Frontier Science, The University of Tokyo, Chiba, Japan; 3 Core Research for Evolutional Science and Technology (CREST), Japan Science and Technology Agency, Tokyo, Japan; 4 Department of Cell Signaling, Graduate School of Medical and Dental Sciences, Tokyo Medical and Dental University, Tokyo, Japan; 5 Laboratory of Developmental Genetics, The Institute of Medical Science, The University of Tokyo, Tokyo, Japan; 6 Department of Immunology, Graduate School of Medicine and Faculty of Medicine, The University of Tokyo, Tokyo, Japan; University of Vermont, United States of America

## Abstract

The Runt-related transcription factor 2 (*Runx2*) gene encodes the transcription factor Runx2, which is the master regulator of osteoblast development; insufficiency of this protein causes disorders of bone development such as cleidocranial dysplasia. Runx2 has two isoforms, Runx2-II and Runx2-I, and production of each isoform is controlled by a unique promoter: a distal promoter (P1) and a proximal promoter (P2), respectively. Although several studies have focused on differences and similarities between the two Runx2 isoforms, their individual roles in bone formation have not yet been determined conclusively, partly because a *Runx2-I*-targeted mouse model is not available. In this study, we established a novel *Runx2*-manipulated mouse model in which the first ATG of *Runx2-I* was replaced with TGA (a stop codon), and a neomycin-resistant gene (*neo*) cassette was inserted at the first intron of *Runx2-I*. Homozygous *Runx2-I^neo/neo^* mice showed severely reduced expression of *Runx2-I*, whereas *Runx2-II* expression was largely retained. *Runx2-I^neo/neo^* mice showed neonatal lethality, and in these mice, intramembranous ossification was more severely defective than endochondral ossification, presumably because of the greater involvement of Runx2-I, compared with that of Runx2-II in intramembranous ossification. Interestingly, the depletion of *neo* rescued the above-described phenotypes, indicating that the isoform-specific N-terminal region of Runx2-I is not functionally essential for bone development. Taken together, our results provide a novel clue leading to a better understanding of the roles of Runx2 isoforms in osteoblast development.

## Introduction

More than 15 years have passed since the Runt-related transcription factor 2 (*Runx2*) gene was identified as the master regulator of bone development and osteoblast differentiation [1], [2]. Mutation of *Runx2* causes cleidocranial dysplasia; patients with this condition, many of whom have an inherited heterozygotic mutation in the coding region of the gene, exhibit defective endochondral and intramembranous ossification [3]. Extensive research has shed light on the role of Runx2 in the development of the mineralized bone skeleton and on how Runx2 orchestrates interactions between the array of other molecules involved in the regulation of skeletal development.

The Runx family of transcription factors comprises three members (Runx1, Runx2, and Runx3), all of which have a conserved Runt homology domain that recognizes a specific nucleotide motif (R/TACCRCA) and works in tissue-specific ways [4]. The *Runx1* gene undergoes chromosomal translocation that results in the formation of a fusion protein (Runx1-ETO) associated with myeloid and lymphoid malignancies [5]. In addition, Runx proteins were recently discovered to exhibit transactivation and cooperative functions in conjunction with the Foxp3 transcription factor in regulatory T cells [6], [7]. Loss of function of Runx3 in epithelial cells is strongly associated with hyperplasia [4].

Each of the *Runx* genes has at least two major splicing variants [4]. In the case of *Runx2*, a distal promoter (P1) leads to the production of Runx2-II (also known as P1Runx2), and a proximal promoter (P2) induces the production of Runx2-I (also known as P2Runx2). The amino acid sequence of Runx2-I shows>99% similarity to that of Runx2-II; the two isoforms share a common 509-amino-acid sequence, but Runx2-I has a distinctive 5-amino-acid N-terminal sequence that differs from the 19-amino-acid N-terminal sequence of Runx2-II [8], [9]. Runx2-II is predominantly expressed in bone tissue, whereas Runx2-I is produced both in bone and in other tissues such as liver and lung tissue [8], [10]. However, the association between the distinct N-terminal amino acid compositions of the two isoforms and their respective functions in bone development is not well understood.

Various investigators have suggested two hypotheses for understanding Runx2 function. One hypothesis is that the two isoforms play different roles in bone development. For example, using an *in situ* hybridization method with probes that specifically recognize *Runx2* variants, Park and colleagues showed that the two *Runx2* splicing variants are expressed in distinctive ways at the site of cranial suture formation [11]. The other hypothesis is that the functioning of Runx2 depends on the total dose of protein. For example, Zhang *et al*. reported that the severity of the insufficient-bone-formation phenotype increases dose-dependently with the total amount of Runx2 protein, as indicated by experiments in which they intercrossed two lines of Runx2-deficient mice, one lacking both Runx2-II and Runx2-I and the other lacking only Runx2-II [12]. Note that these two hypotheses are not mutually exclusive. Taken together, the results of previous experiments suggest that the two Runx2 isoforms work in cooperative and distinctive ways and that their fates are determined by spatial and temporal regulation. Unification of these two hypotheses will require additional studies, especially studies focusing more directly on the function of Runx2-I. However, an appropriate mouse model is unavailable.

In this work, we established a novel Runx2-deficient mouse model with a *neo* cassette inserted into the second intron of the *Runx2* gene and with a mutation of the translation start codon (ATG) of *Runx2-I* into a stop codon (TGA). In homozygous mice, *Runx2-I* expression was reduced by 84% relative to that in wild-type mice, and more than 40% of *Runx2-II* expression was retained. Consequently, the homozygous mice showed 67% reduction of total *Runx2* expression and died at the early postnatal stage. Morphological and quantitative analyses of skeletal bones revealed that dysfunction in intramembranous ossification was more severe than dysfunction in endochondral ossification. These developmental defects were the consequence of inefficiency of osteoblast differentiation, as evidenced by *in vitro* assays. Importantly, removal of the *neo* cassette completely restored the viability of the gene-targeted mice, suggesting the insignificance of the isoform-specific amino acid sequence of Runx2-I. This study adds new pieces to the puzzle of Runx2 function in bone development.

## Materials and Methods

### Generation of *Runx2-I*-targeted mice

Genomic DNA containing *Runx2* was isolated from E14-1 embryonic stem cells, and the sequence was confirmed by sequencing analysis. A targeting vector was designed to change the first ATG of *Runx2-I* to TGA (a stop codon). A total of 30 µg of linearized vector was electroporated into E14-1 embryonic stem cells, which were then subjected to positive selection by G418 and negative selection by ganciclovir. Homologous recombination in the selected cells was confirmed by polymerase chain reaction (PCR) and Southern blot analyses. The selected clones were then microinjected into blastocysts of C57BL/6 mice. Mating of chimeric male mice to C57BL/6 female mice resulted in the transmission of the mutant allele to the germline. For bone morphological analyses, F1 mice were used. To evaluate gene expression levels, we used mice backcrossed with C57BL/6 at least six times. All animals were housed under specific pathogen–free conditions at the animal facility of the Institute of Medical Science, the University of Tokyo. All animal experiments were done with the approval of the Animal Research Committee of The Institute of Medical Science, The University of Tokyo.

### Morphological analyses

Pregnant mice were euthanized by cervical dislocation at 18.5 days postcoitum (dpc), and fetuses were euthanized by decapitation. All organs except for the brain were removed, and skin was excised carefully; then the fetuses were fixed and dehydrated in 100% ethanol overnight. Specimens were first stained with Alcian blue solution for 1 day; after lysis of the remaining skin and remnants of muscle and other organs with 2% KOH, the specimens were stained with Alizarin red solution. The stained skeletons were transferred to glycerol for preservation and observation.

### Cell cultures

Cells were collected from the calvaria of fetuses as follows [13]. Pregnant mothers were euthanized at 18.5 dpc, and fetuses were euthanized. Skull bones were extracted and digested (five times, 10 min each time) in minimum essential medium alpha (α-MEM) containing 10% fetal calf serum, 10 unit/ml penicillin, 10 µg/ml streptomycin, 0.1% collagenase (Wako, Osaka, Japan), and 0.2% dispase (Roche, Indianapolis, IN). The supernatant from the first 10-min digestion was discarded. Cells obtained from the remainder of the digestions were pooled and seeded with normal α-MEM for expansion for 3 days.

For osteoblast differentiation, we followed the method described previously [13], [14]. Briefly, 4×10^5^ cells were seeded in 24-well culture plates and cultured for 2 weeks in the osteogenic medium (α-MEM supplemented with 50 µM ascorbic acid, 10 nM dexamethasone, and 10 mM β-glycerophosphate). Alizarin red staining was performed as previously described [14]. Briefly, after the removal of the culture medium, cells were washed twice with PBS and fixed with 4% paraformaldehyde for 10 min. Washing of the culture plate with PBS was followed by incubation with Alizarin red solution overnight at room temperature. Finally, plates were washed with pure water and dried. The number of bone nodules was counted. The alkaline phosphatase assay was conducted according to the manufacturer’s instructions (Wako).

### Quantitative real-time PCR

Total RNA was extracted from calvarial cells by TRIzol reagent (Invitrogen, Carlsbad, CA). RNA was reverse transcribed by SuperScriptIII (Invitrogen) with an Oligo(dT) primer or a random primer (Invitrogen), and then template RNA was digested with RNase H. Quantitative PCR was performed with SYBR Green (Roche) with specific primers for each gene; the primers were designed to flank at least one intron. The specificity of the primers was confirmed by amplicon sequencing. Respective *Runx2* variants were amplified by using three forward primers and one reverse primer; 5′-CAGCGCAGTGACACCGTGTCAGC-3′ (*Runx2-II* forward), 5′-GGCCACTTCGCTAACTTGTGG-3′ (*Runx2-I* forward), 5′-GCGGTGCAACAAGACCCTG-3′ (forward common), 5′-CCGGCCATGACGGTAACC-3′ (reverse common). For each of the *Runx2* variants, samples were amplified for 45 cycles of 10 s at 95°C followed by 10 s annealing at 65°C followed by 30 s of extension at 72°C. For the common sequence of *Runx2*, samples were amplified for 45 cycles of 10 s at 95°C followed by 10 s annealing at 60°C followed by 10 s extension at 72°C. The primers used were as follows: *Alpl*, 5′-CGGATCCTGACCAAAAACC-3′ and 5′-TCATGATGTCCGTGGTCAAT-3′; *Bglap*, 5′-AGACTCCGGCGCTACCTT-3′ and 5′-CTCGTCACAAGCAGGGTTAAG-3′; *Col1a1*, 5′-CATGTTCAGCTTTGTGGACCT-3′ and 5′-GCAGCTGACTTCAGGGATGT-3′; *Tnfsf11*, 5′-TGAAGACACACTACCTGACTCCTG-3′ and 5′-CCACAATGTGTTGCAGTTCC-3′; *Sp7*, 5′-CGTCCTCTCTGCTTGAGGAAG-3′ and 5′-GCCGCCAAATTTGCTGCAGG-3′; housekeeping gene *Gapdh*, 5′-TGTCCGTCGTGGATCTGAC-3′ and 5′-CCTGCTTCACCACCTTCTTG-3′. For amplification of these gene fragments, samples were amplified for 45 cycles of 10 s at 95°C followed by 10 s annealing at 60°C followed by 10 s extension at 72°C.

### Cloning, transfection and immunoblotting

The coding regions for Runx2-II and Runx2-I, each with an endogenous Kozak sequence, were amplified by standard reverse transcription (RT)-PCR using total RNA prepared from calvarial cells. The confirmed sequence was inserted into pcDNA3.1(+) expression vector (Invitrogen) by using the *BamH* I and *Xho* I sites. Each expression vector was used for transfection of HEK293 cells by Lipofectamine 2000 (Invitrogen). Nuclear extracts were prepared from transfected HEK293 cells or primary calvarial cells with an NE-PER Nuclear and Cytoplasmic Extraction Reagent Kit (Thermo, Rockford, IL) according to the manufacturer’s instructions. Nuclear extracts were separated by NuPAGE 4–12% Bis-Tris Gel (Invitrogen) and were transferred onto a polyvinylidene difluoride membrane. The membrane was blotted with anti-Runx2 (Clone 8G5; MBL, Nagoya, Japan) and anti-Lamin A/C (clone 14/LaminAC; BD Biosciences, Oxford, UK) monoclonal antibodies. Proteins on membranes were visualized with an ECL Prime Western Blotting Detection System and an ImageQuant LAS 4000 imager (GE Healthcare, Uppsala, Sweden).

### Statistical analysis

Data are given as means ± standard error of the mean (SEM). Student’s *t*-test was performed for experiments conducted with the same number of samples for each group. Otherwise, we used the Mann–Whitney *U*-test for statistical analyses when it was needed. Differences were considered to be statistically significant at *p* values of <0.05: *p<0.05, **p<0.01.

## Results

### Establishment of a line of Runx2-I-deficient mice

**Figure 1:**
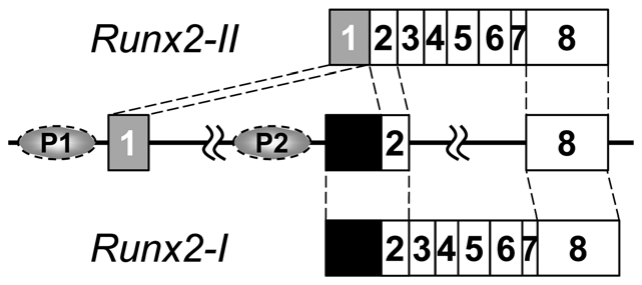
*Runx2* gene construct and splicing patterns. *Runx2* transcription is induced by distal and proximal promoters (P1 and P2, respectively). Although the first exon of *Runx2-II* contains a 197-bp mini-intron, the two parts of the exon are often shown as a single exon (exon 1), as we have done here (gray box). Exon 2, the first exon for *Runx2-I* consists of a *Runx2-I*-specific sequence (black box; includes a coding sequence that is translated into five amino acids unique to Runx2-I, MRIPV) and a common sequence (white box; corresponds to the second exon of *Runx2-II*).

**Figure 2:**
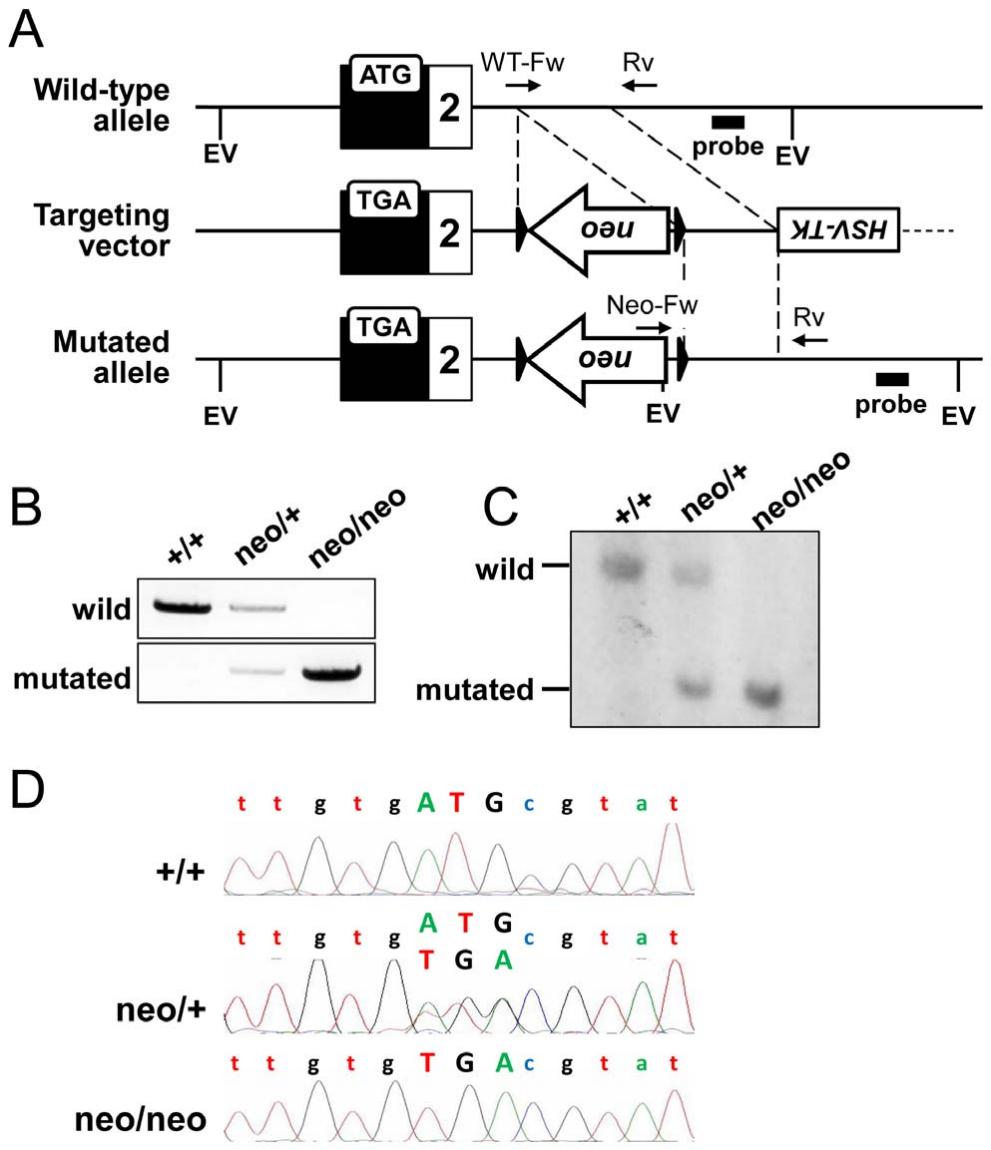
Generation of *Runx2-I*-targeted mice. (A) Schematic representation of the targeting strategy. A targeting construct was designed to replace the start codon (ATG) of *Runx2-I* with a stop codon (TGA). Relevant *Eco*R V (EV) recognition sites are indicated. (B) PCR analysis of the indicated *Runx2-I*-targeted mice. The primers depicted in panel (A) were used to detect the wild-type allele (WT-Fw and Rv) or the mutated allele (Neo-Fw and Rv). The PCR products were 1.1 kb in size for both the wild-type and the mutated allele. +/+, wild-type; neo/+, heterozygous; neo/neo, homozygous. (C) Confirmation of recombination by Southern blot analysis using the genome of *Runx2-I*-targeted littermates after digestion by *Eco*R V. The membrane was blotted with the probe shown in panel (A). The size of the band was 7.2 kb in the wild-type allele and 2.2 kb in the mutated allele. (D) Sequence analysis of the first ATG of *Runx2-I* in the mice of each genotype. The genome sequence was amplified by using the set of primers flanking the first ATG region, and the amplified fragment was read by the DNA sequencer.

The extreme 3′ end of the first exon of *Runx2-I* corresponds to exon 2 of *Runx2-II* (Fig. 1); therefore, to generate a mouse line with deficient Runx2-I expression, we constructed a targeting vector by mutating the first ATG of *Runx2-I* to a stop codon (TGA) to interrupt Runx2-I translation without introducing any changes to the coding sequence for Runx2-II (Fig. 2A). Using homologous recombinant embryonic stem cells, we obtained germline-transmitted chimeric mice, as well as heterozygous descendants (*Runx2-I^neo/+^*). Because *Runx2*-null mice die soon after birth [1], [2], we confirmed on the day of birth (P0) that the expected gene manipulation occurred in the offspring of heterozygote intercrosses (Fig. 2B). Homologous recombination with the target gene in offspring was also confirmed by Southern blot analysis (Fig. 2C). The non-existence of undesired random recombination was confirmed by Southern blotting with a probe against the *neo* cassette (data not shown). In addition, DNA sequencing confirmed the mutation of the first ATG of *Runx2-I*; equal-intensity signals for ATG and TGA were observed in *Runx2-I^neo/+^* mice, whereas only the TGA signal was observed in *Runx2-I^neo/neo^* mice (Fig. 2D). Although heterozygote intercrossing was continued until the number of pups totaled 100, we found no homozygous mice in a 3-week-old litter (*Runx2-I^+/+^* 44; *Runx2-I^neo/+^* 68; *Runx2-I^neo/neo^* 0). Note, we identified live homozygous embryos on embryonic day 18.5 in normal Mendelian ratios. However, all homozygous mice died within 24 h after birth. These data are quite similar to data for *Runx2*-null mice [1], [2].

### Runx2-I expression was severely diminished in *Runx2-I* ^neo/neo^ mice but not in *Runx2-I* ^TGA/TGA^ mice

**Figure 3:**
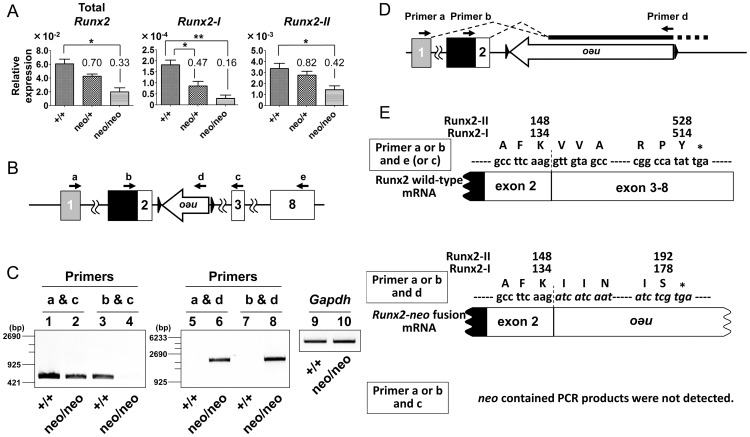
The *neo* transgene perturbed *Runx2* expression. (A) Expression of total *Runx2*, *Runx2-I*, and *Runx2-II* in *Runx2-I*-mutated mice. Expression was analyzed by quantitative real-time PCR using RNA prepared from E18.5 calvarial cells of wild-type (+/+), *Runx2-I^neo/+^* (neo/+) and *Runx2-I^neo/neo^* (neo/neo) mice. The results were normalized against the expression of *Gapdh*. Data are representative of three independent experiments and are means ± SEM (*n* = 3/group); *p<0.05, **p<0.01. (B) PCR primers (arrows) were placed on *Runx2* exons and the *neo* transgene. (C) Semiquantitative RT-PCR analysis of RNA prepared from E18.5 calvarial cells of wild-type (+/+) and *Runx2-I^neo/neo^* (neo/neo) mice by using the primer set depicted in panel (B). *Gapdh* was simultaneously amplified as an internal control (lanes 9 and 10). (D) Schematic diagram of alternative splicing pattern for *Runx2*-*neo* fusion transcripts. (E) Sequences of the fusion transcripts and proteins. The nucleotide sequence shown in italics is from *neo*, and the asterisk indicates the stop codon in the reading frame. The amino acids corresponding to each codon, and their numbers in the sequence of each Runx2 isoform are also indicated.

Next, we determined whether the expression of *Runx2-I* was affected in *Runx2-I^neo/neo^* mice, in which the only mutation was the replacement of the first ATG (Fig. 2A). Heterozygous mice were intercrossed to obtain embryonic (18.5 dpc) littermates. Calvarial cells were prepared from skull bones of *Runx2-I^+/+^*, *Runx2-I^neo/+^*, and *Runx2-I^neo/neo^* embryos by means of enzymatic digestion. Quantitative real-time PCR was performed to determine total *Runx2* expression and expression of the individual variants. Unexpectedly, total *Runx2* expression and expression of *Runx2-I* was significantly diminished in *Runx2-I^neo/neo^* mice (Fig. 3A). Furthermore, *Runx2-II* expression was also reduced in these mice, although the reduction was more prominent for the *Runx2-I* transcript.

Several previous reports showed that *neo* insertion into an intron results in production of aberrant chimeric transcripts derived from the target gene and *neo* [15]–[18]. Therefore, we assumed that the aberrant transcripts interfered with transcription of both *Runx2-II* and *Runx2-I*. To test this assumption, we performed semiquantitative RT-PCR followed by DNA sequencing by using RNA samples prepared from E18.5 embryos and primers designed for *Runx2* and *neo* (Fig. 3B). When we set primers on the first exon of each gene (exon 1 for *Runx-II* [primer a] and exon 2 for *Runx2-I* [primer b]) and on exon 3 (primer c) to amplify *Runx2-II* or *Runx2-I* genes, we detected PCR products of approximately 500 bp, which were derived from normally spliced RNA, in samples from wild-type mice (Fig. 3C, *lanes 1* and *3*; Fig. 3E, *upper panel*). Consistent with the results from quantitative real-time PCR, amplicons of both *Runx2* variants, especially *Runx2-I*, were decreased in samples from *Runx2-I^neo/neo^* mice (Fig. 3C, *lanes 2* and *4*). In addition, although some previous reports indicated that *neo* inserts into the targeted gene transcripts [15]–[17], we did not observe fusion transcripts formed in this way in this experiment (Fig. 3C, *lanes 1–4*; Fig. 3E, *lower panel*). Therefore, we next set a reverse primer on the promoter region of the *neo* cassette (primer d) and performed the same experiments. Whereas no amplicons were detected from wild-type samples (Fig. 3C, *lanes 5* and *7*), one PCR product was observed in samples from *Runx2-I^neo/neo^* mice from both primer sets (Fig. 3C, *lanes 6* and *8*). DNA sequencing of these amplicons revealed that there were at least two alternatively and abnormally spliced RNAs between exon 1 or 2 and *neo* in *Runx2-I^neo/neo^* mice (Fig. 3D and E, *middle panel*). Although we could not define the 3′-end of these *Runx2*-*neo* fusion RNAs, translation of these transcripts gave just C-terminal-truncated Runx2 protein fused with artificial peptides and lacking a nuclear localization signal and most of the Runt homology/DNA-binding domain (Fig. 3E, *middle panel*). Taken together, these data suggest that insertion of the *neo* cassette into an intron between exons 2 and 3 affected normal *Runx2* expression at the transcriptional level.

**Figure 4:**
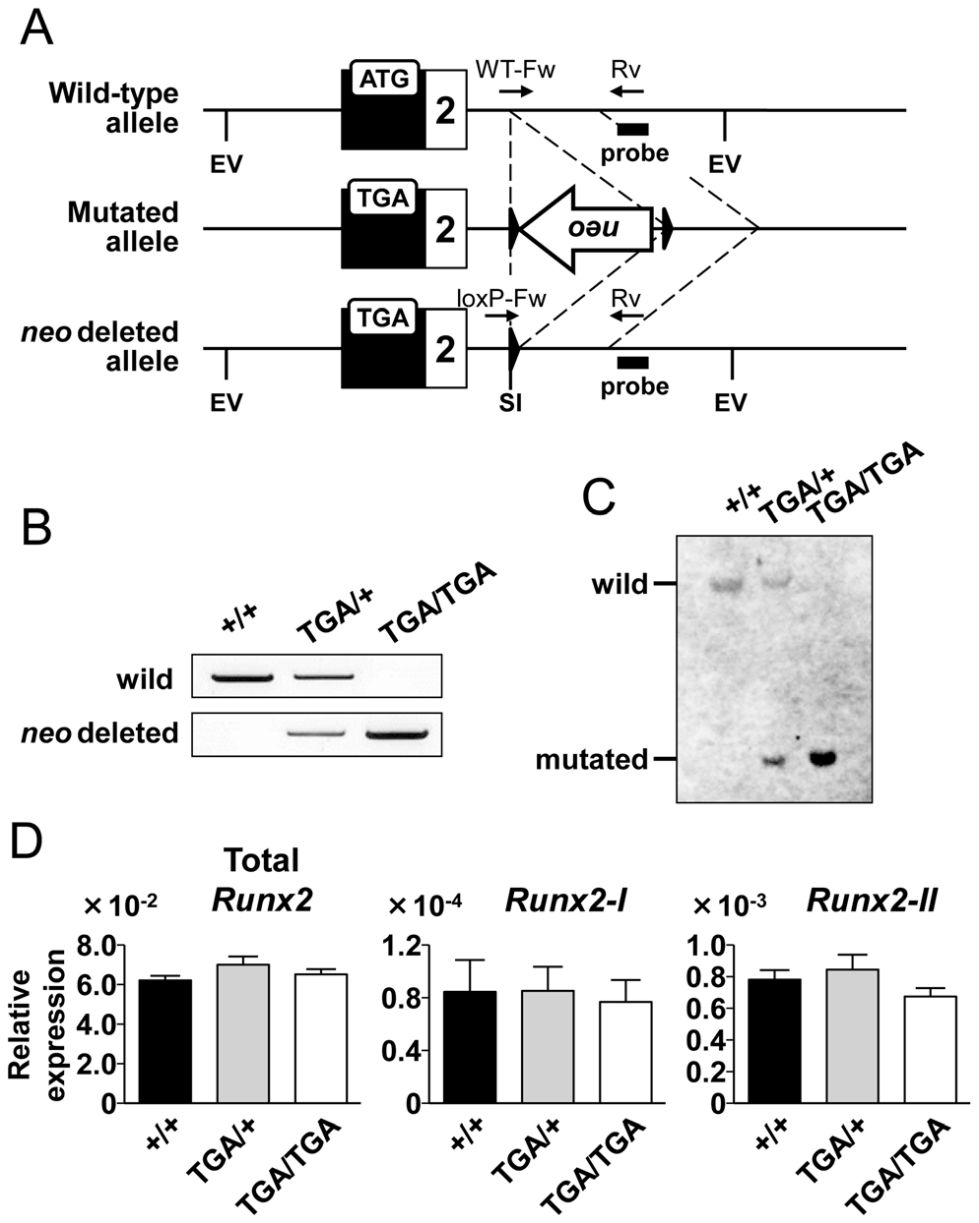
The lethality in *Runx2-I^neo/neo^* mice was rescued by *neo* cassette deletion. *Runx2-I^neo/+^* mice crossed with a Cre deleter mice to remove the *neo* cassette. (A) Schematic illustration of depletion of the *neo* cassette. Relevant *Eco*R V (EV) and *Sal* I (SI) recognition sites are indicated. (B) Deletion of the *neo* cassette was confirmed by PCR. The primers depicted in panel (A) were used to detect the wild-type allele (WT-Fw and Rv) and the *neo*-deleted allele (loxP-Fw and Rv). The PCR products were 1.1 kb in size for both the wild-type and the *neo*-deleted allele. +/+, wild-type; TGA/+, heterozygous; TGA/TGA, homozygous. (C) Southern blot analysis using the genome of the indicated *Runx2-I*-targeted littermates after digestion by *Eco*R V and *Sal* I. The membrane was blotted with the probe shown in panel (A). The size of the band was 7.2 kb in the wild-type allele and 1.9 kb in the *neo*-deleted allele. (D) Fetal calvarial cells were prepared from generated mice. RNA was purified from mice of each genotype and then analyzed by quantitative real-time PCR for expression levels of total *Runx2*, *Runx2-I*, and *Runx2-II*. Data are representative of three independent experiments and are means ± SEM (*n* = 3/group).

To more directly check the influence of *neo* cassette insertion, we excised the cassette by crossing *Runx2-I^neo/+^* mice with Cre deleter mice (Fig. 4A). The complete depletion of *neo* was confirmed by PCR (Fig. 4B), Southern blot analysis (Fig. 4C), and gene sequencing (data not shown). Interestingly, the survival of homozygous (*Runx2-I^TGA/TGA^*) mice was comparable to that of wild-type littermates (*Runx2-I^+/+^* 27; *Runx2-I^TGA/+^* 48; *Runx2-I^TGA/TGA^* 27), suggesting that the lethality observed in *Runx2-I^neo/neo^* mice was completely rescued by *neo* cassette deletion. This interpretation was supported by quantification of individual *Runx2* gene transcripts: the amounts of the *Runx2* variant transcripts and total *Runx2* transcripts were comparable among the calvarial cells of mice of the three genotypes (Fig. 4D). These data indicate that the expression of the *Runx2* gene was compromised by *neo* cassette insertion in *Runx2-I^neo/neo^* mice.

**Figure 5:**
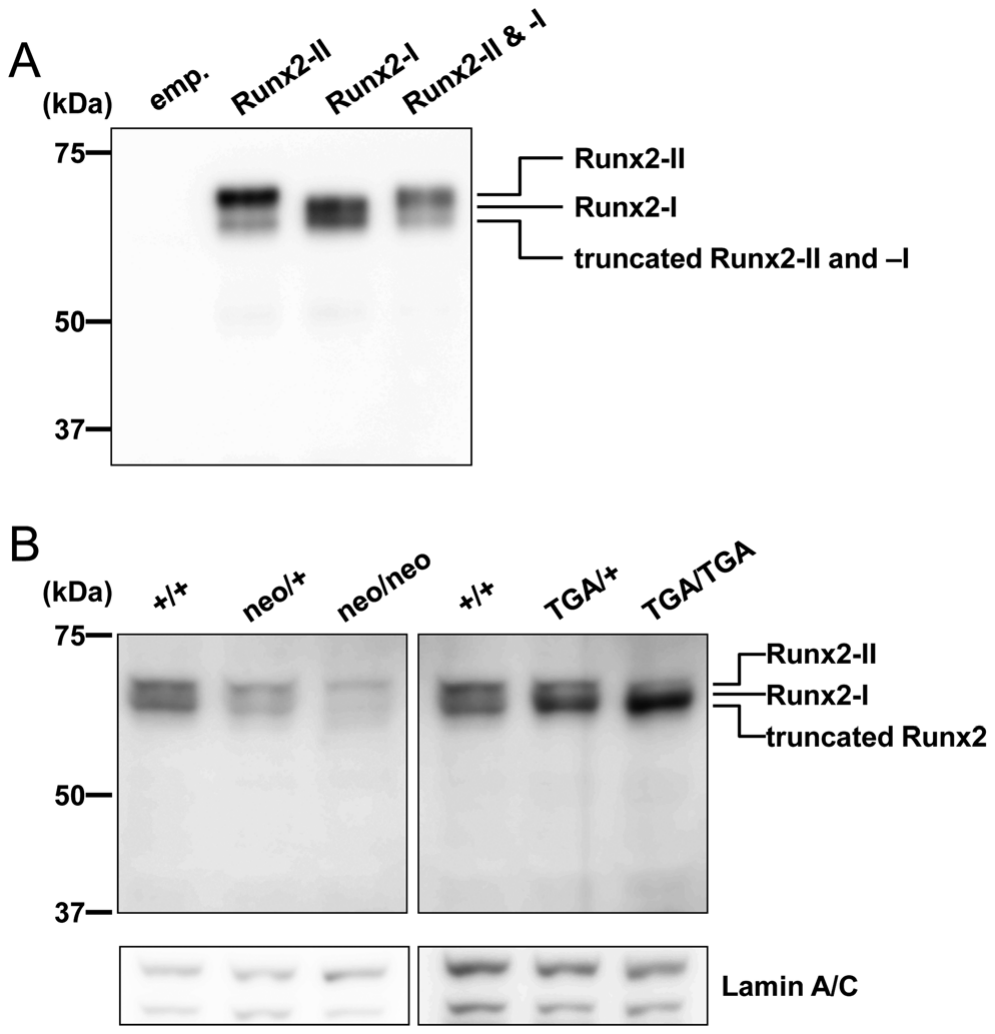
Runx2-I was expressed in *Runx2-I^TGA/TGA^* mice as N-terminal truncated Runx2. (A) Anti-Runx2 mAb (clone: 8G5) could recognize Runx2-II, Runx2-I, and their truncated versions. HEK293 cells were transiently transfected with the indicated expression vectors (see Materials and Methods). Forty-eight hours after transfection, nuclear proteins were obtained from the cells and then immunoblotted with anti-Runx2 mAb. (B) Nuclear proteins of calvarial cells freshly prepared from embryos (18.5 dpc) with the genotypes indicated in the figure were immunoblotted with anti-Runx2. The membranes were also blotted with anti-Lamin A/C mAb as an internal control. The data are representative of three independent experiments.

Note that although the *neo*-depleted *Runx2-I^TGA/TGA^* mice still had the point mutation at the translation start codon (ATG to TGA), they showed no lethality, as described above. Therefore, we examined the expression level of each Runx2 isoform in *Runx2-I^neo/neo^* and *Runx2-I^TGA/TGA^* mice by means of a series of immunoblot analyses. It has been reported that clone 8G5 anti-Runx2 monoclonal antibody (mAb) is able to detect endogenous mouse Runx2 protein [12], [19]. However, defining which band is from which isoform was difficult in previous studies. Thus, we first checked whether this mAb could recognize both Runx2 isoforms. HEK293 cells were transfected with the expression vector for each Runx2 isoform individually or with both vectors, and then nuclear extracts were prepared for immunoblotting. All samples showed one major band of the predicted size, and one band for a truncated protein, which was the same size for all the samples (Fig. 5A, upper and lower bands, respectively), suggesting that the lower band was derived from N-terminal truncation. In addition, the truncated Runx2 seemed to be preferentially derived from *Runx2-I* transcripts rather than from *Runx2-II* transcripts.

Next, we evaluated Runx2 expression in the *Runx2-I*-manipulated calvarial cells at 18.5 dpc. Consistent with the quantitative real-time PCR results, the Runx2-I signal, but not the Runx2-II, signal was severely decreased in *Runx2-I^neo/neo^* mice (Fig. 5B, *left panel*). In contrast, calvarial cells from *neo*-depleted *Runx2-I^TGA/TGA^* embryos did not exhibit any decrease in the Runx2 signal (Fig. 5B, *right panel*). To the contrary, the signal density of the lower band was increased in the cells from homozygous mice, indicating the accumulation of truncated Runx2-I protein in these mice. These results clearly indicate that N-terminal-truncated Runx2-I was produced in *Runx2-I^TGA/TGA^* mice even in the absence of the first ATG. This result also supports the idea that the Runx2-I-specific amino acid sequence (MRIPV) has little or no importance for the function of the isoform.

### Expression of osteoblast marker genes was impaired in *Runx2-I* ^neo/neo^ calvarial cells

**Figure 6:**
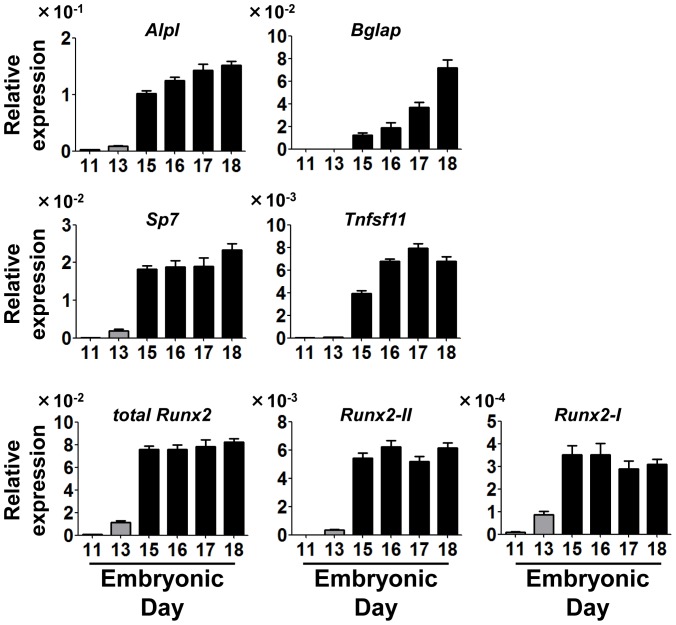
Osteoblastic gene expression profile during fetal development. Pregnant C57BL/6 mice were euthanized for examination of fetuses on days 11, 13, 15, 16, 17, and 18 postcoitum. The calvaria of fetuses were enzymatically digested, and collected cells were subjected to the quantitative real-time PCR analyses. Because the fetuses at days 11 and 13 did not yet have firm calvarial structures, we instead used the upper part of head tissue, which might have contained cells other than calvarial cells (e.g., brain and skin cells). Data are means ± SEM (*n* = 7–9/group).

**Figure 7:**
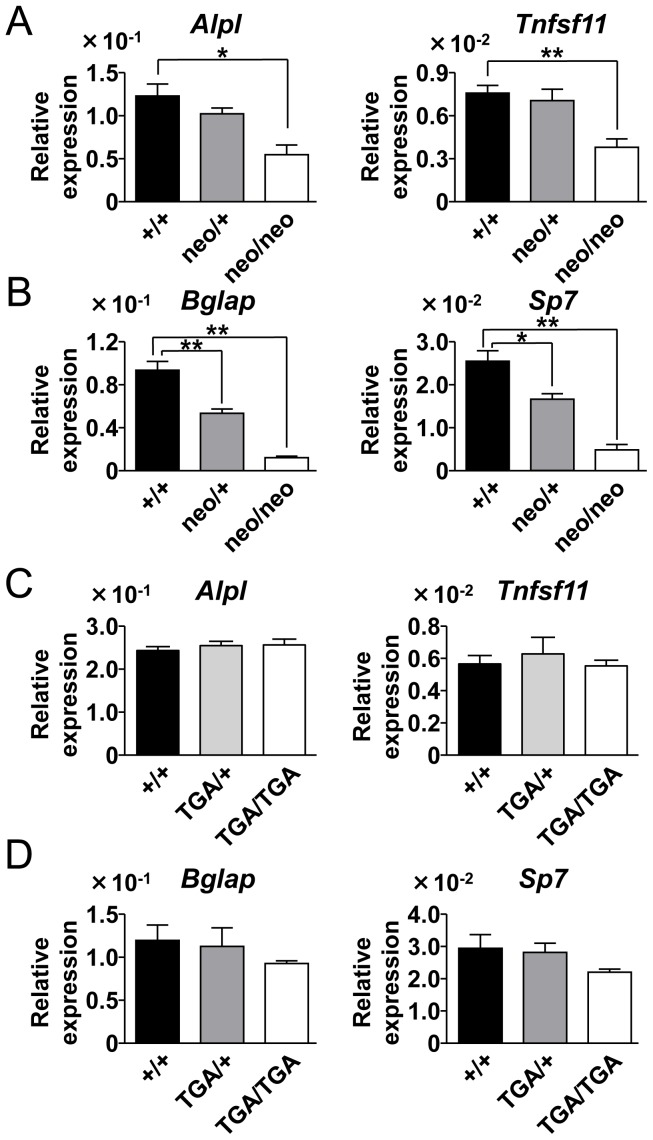
Osteoblastic gene expression was diminished in *Runx2-I^neo/neo^* calvarial cells. Expression of the indicated osteoblastic genes in calvarial cells (A and C) and osteoblast differentiated cells (B and D) from *Runx2-I*-mutated (A and B; +/+, neo/+, neo/neo, C and D; +/+, TGA/+, TGA/TGA). Quantitative real-time PCR analysis was performed by using the same RNA used for the analyses shown in Figure 3A (*neo*-possessing mice) and Figure 4D (*neo*-deleted mice). Data are representative of three independent experiments and are means ± SEM (*n* = 3/group); *p<0.05, **p<0.01.

Because neonatal lethality is the most notable phenotype reported for Runx2-deficient mice, which are unable to breathe, presumably because of severe malformation of the sternum and the rib [1], [2], we determined whether bone formation in *Runx2-I^neo/neo^* mice was delayed compared with that in littermate controls. We quantified the levels of marker genes for osteoblast development by means of quantitative real-time PCR. Using C57BL/6 mice, we confirmed in advance the up-regulation of all genes examined in this experiment during the embryonic stage (Fig. 6). Among these genes, the expression levels of *Alpl* (which encodes alkaline phosphatase) and *Tnfsf11* (which encodes RANKL) were significantly decreased in *Runx2-I^neo/neo^* mice compared with the levels in wild-type controls (Fig. 7A). In contrast, expression of *Bglap* (which encodes osteocalcin) and *Sp7* (which encodes osterix), expression of which is reportedly diminished in *Runx2-II*-knockout mice [20], did not change significantly at the time of fresh isolation of calvarial cells (data not shown). However, after 14-day culture for osteoblast differentiation, expression of *Bglap* and *Sp7* was significantly impaired in the cells obtained from homozygous mice compared with expression in cells from wild-type mice (Fig. 7B), indicating that bone development in *Runx2-I^neo/neo^* mice was severely affected. Calvarial cells from *neo*-depleted *Runx2-I^TGA/TGA^* mice were also analyzed by quantitative PCR, and the expression level of *Alpl* and *Tnfsf11* at the time of cell preparation and the expression levels of *Bglap* and *Sp7* after 14-day culture under osteoblast differentiating conditions were all comparable between the gene-manipulated mice and littermate controls (Fig. 7C and D). Thus, *neo* insertion into the second intron of the *Runx2* gene and resulting severe impairment of *Runx2-I* expression caused fatal abrogation of bone-related-gene expression, but the insufficiency in expression was totally canceled by *neo* removal, even though the endogenous start codon of *Runx2-I* had been mutated.

### *Runx2-I* ^neo/neo^ mice showed morphologically delayed bone formation

**Figure 8:**
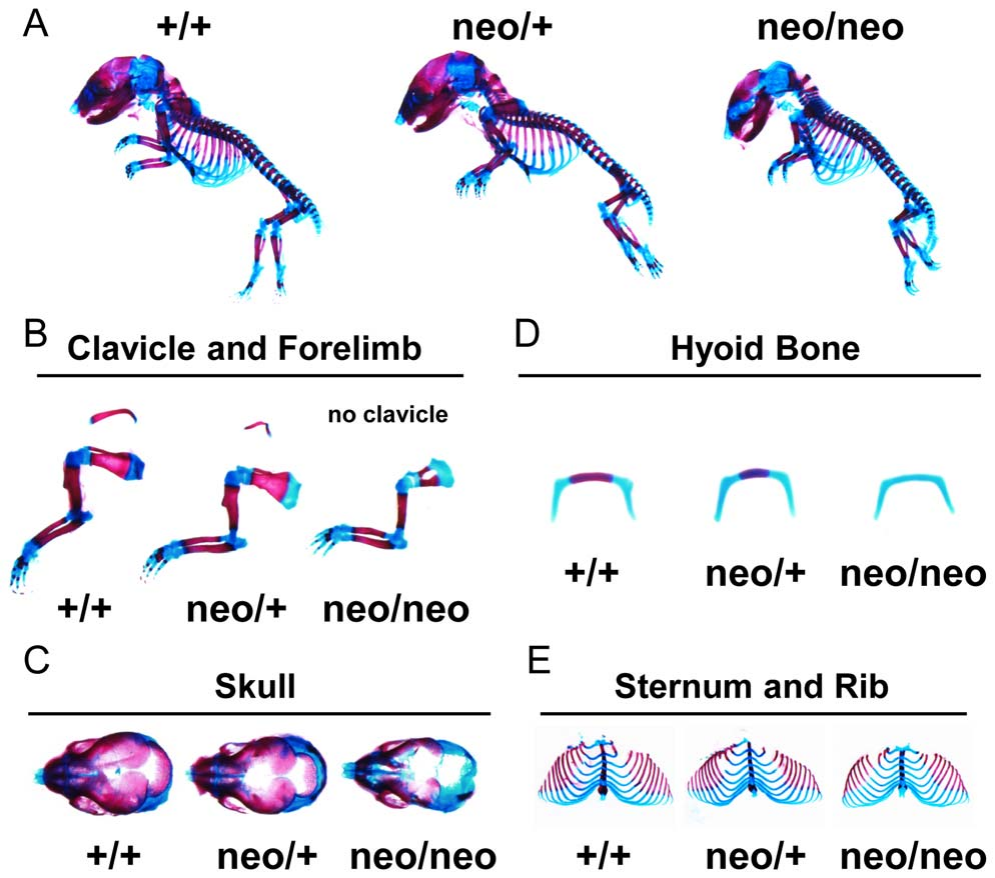
*Runx2-I^neo/neo^* mice showed severely delayed bone ossification. Skeletal tissues of E18.5 fetuses of wild-type (+/+), *Runx2-I^neo/+^* (neo/+) and *Runx2-I^neo/neo^* (neo/neo) mice were stained with Alcian blue to detect cartilage, and Alizarin red to detect calcified bone. After whole-body images were captured (A), specimens were separated into parts: (B) clavicle and forelimb; (C) skull; (D) hyoid bone; (E) sternum and rib.

To evaluate ossification in *Runx2-I^neo/neo^* mice, we conducted whole-body bone staining with Alizarin red and Alcian blue. Unlike *Runx2*-null mice, which show complete loss of ossified bone [1], *Runx2-I^neo/neo^* mice displayed diminished ossification, similar to that observed in *Runx2^+/−^* mice (Fig. 8A-E), even though their neonatal lethality was similar to that of *Runx2*-null mice [1], [2]. In addition, *Runx2-I^neo/neo^* mice exhibited more-severe bone loss than Runx2-II-deficient mice [20], as indicated by complete loss of the clavicle in the former (Fig. 8B). Furthermore, *Runx2-I^neo/neo^* mice lacked the occipital bone, closure of their sagittal suture was severely delayed (Fig. 8C), and complete lack of ossification of hyoid bone was observed (Fig. 8D). Some but not all of the ossification centers were absent in the phalanges of *Runx2-I^neo/neo^* mice (Fig. 8A and B). These results demonstrate hypoplastic skeletal development in *Runx2-I^neo/neo^* mice.

### *Runx2-I^neo/neo^* mice showed reduced bone volume

**Figure 9:**
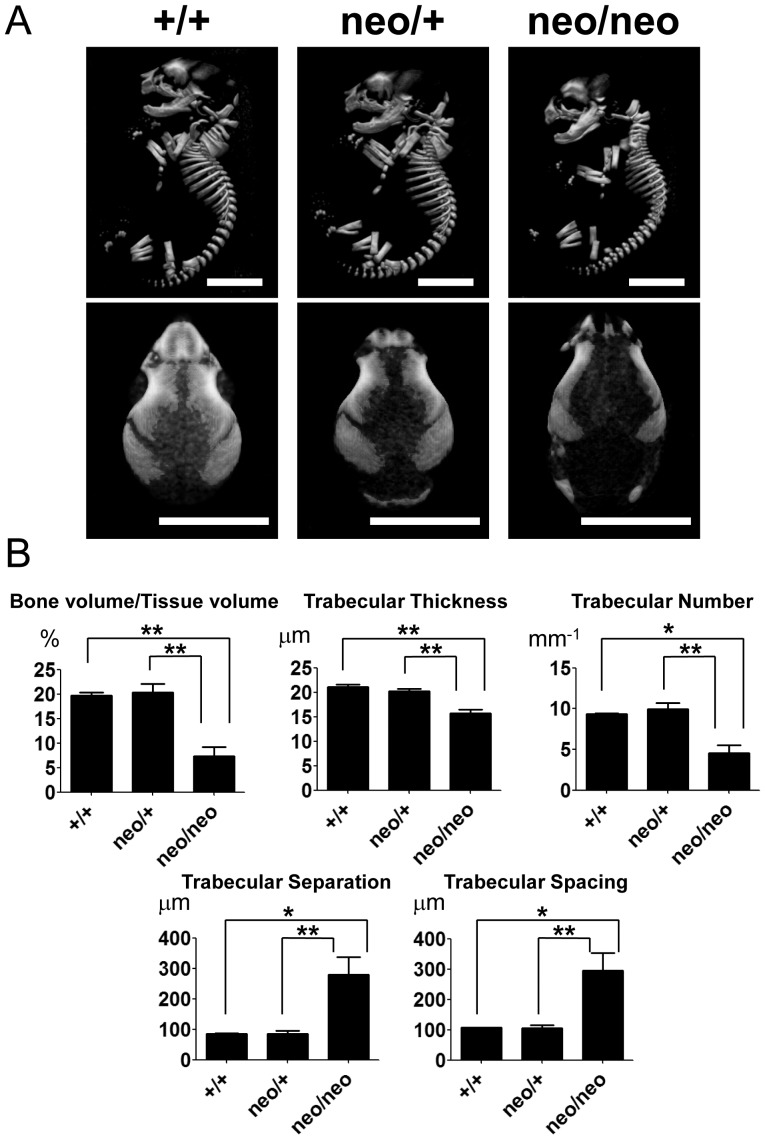
*Runx2-I^neo/neo^* mice showed decreased bone density. (A) Mineralization of E18.5 fetuses of wild-type (+/+), *Runx2-I^neo/+^* (neo/+) and *Runx2-I^neo/neo^* (neo/neo) mice was assessed by microcomputed tomography of the whole body (*upper panels*) and the skull (*lower panels*). Scale bars: 5 mm. (B) Quantitative bone mineral parameters were calculated from femur measurements. All the parameters of *Runx2-I^neo/neo^* mice showed significantly severe deficiency of bone formation. Interestingly, *Runx2-I^neo/+^* mice did not exhibit any abnormalities compared with wild-type littermates. Data are means ± SEM (*n* = 4–9/group); *p<0.05, **p<0.01.

To further quantify defective bone formation in *Runx2-I^neo/neo^* mice, we subjected bones to microcomputed tomography analysis. Computed three-dimensional bone morphology of *Runx2-I^neo/neo^* mice indicated delayed formation of the skeleton, most notable in the calvaria, compared with that of wild-type littermates (Fig. 9A). Consistent with the defective bone formation observed by morphological analyses, bone mass per tissue volume in *Runx2-I^neo/neo^* mice was significantly lower than that in wild-type littermates (Fig. 9B). In addition, the thickness and number of trabecula were decreased, and the trabecular separation and spacing were increased significantly in *Runx2-I^neo/neo^* mice; all these measurements indicate severely delayed ossification in the homozygous mice. Although heterozygous mice showed an intermediate phenotype in morphological analyses of the calvaria (Fig. 9A), the volume and other quantified parameters of their femurs were not statistically different from those of their wild-type littermates.

### Osteoblast development was defective in *Runx2-I* ^neo/neo^ calvarial cells

**Figure 10:**
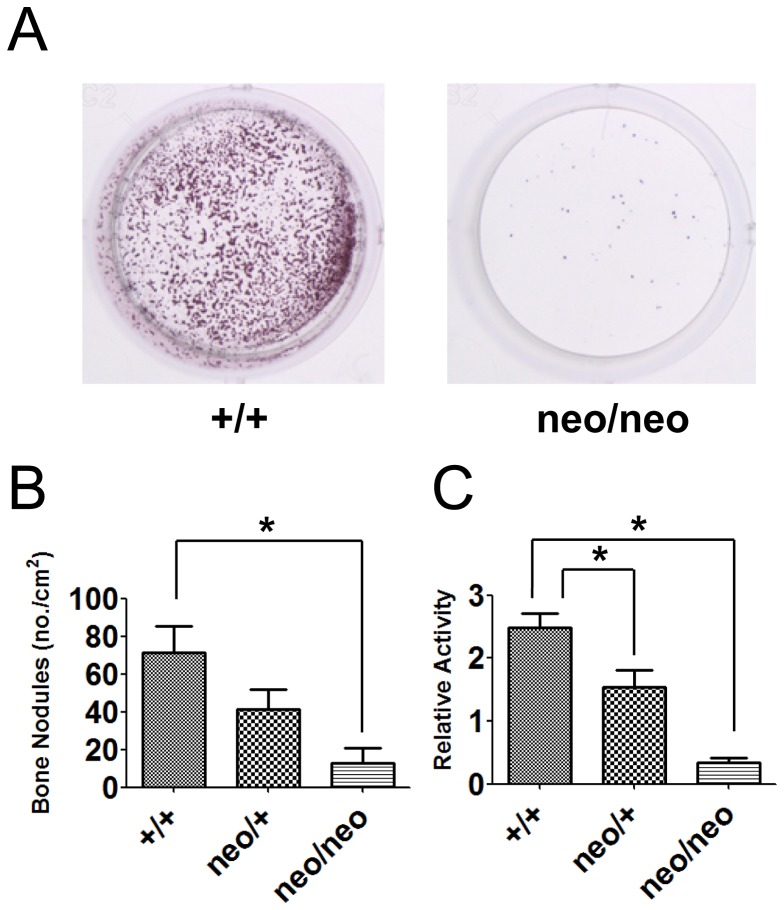
Calvarial cells of *Runx2-I^neo/neo^* mice showed defective osteoblast development. Calvarial cells recovered from wild-type (+/+) and *Runx2-I^neo/neo^* (neo/neo) mice were cultured in osteogenic medium for 14 days and then fixed and stained with Alizarin red to visualize bone nodule formation. Stained culture wells (A) and numbers of bone nodules normalized by culture area (B). Data are means ± SEM (*n* = 2–6/group); *p<0.05. (C) At the same time that the Alizarin red assay was conducted, cells were lysed, and alkaline phosphatase activity was tested. Data are representative of four independent experiments and are means ± SEM (*n* = 3–7/group); *p<0.05.

We speculated that the observed defective bone formation in *Runx2-I^neo/neo^* mice resulted from insufficient osteoblast differentiation. To address this issue, we prepared calvarial cells from fetal mice (18.5 dpc) and cultured the cells in osteoblast differentiation medium for 2 weeks. Cells prepared from *Runx2-I^+/+^* mice developed into osteoblasts, as determined by the massive formation of bone nodules (stained with Alizarin red). In sharp contrast, dramatic reduction of bone nodules was observed in *Runx2-I^neo/neo^* calvarial cells, indicating that these cells could not develop into osteoblasts (Fig. 10A and B). Consistently, alkaline phosphatase activity, measured at the time of bone nodule formation, was also decreased in *Runx2-I^neo/neo^* cells (Fig. 10C). In both of the above-described *in vitro* differentiation assays, heterozygous cells showed a phenotype intermediate between that of the wild-type and the homozygote.

## Discussion

In this study, we established novel *Runx2*-manipulated mouse models: *Runx2-I^neo/neo^* and *Runx2-I^TGA/TGA^*. *Runx2-I^neo/neo^* mice have a *neo* cassette inserted into the second intron of the *Runx2* gene (Fig. 2A). In embryonic day 18.5 calvarial cells from *Runx2-I^neo/neo^* mice, the expression of *Runx2-I* was severely impaired, and *Runx2-II* expression was also affected, albeit to a lesser extent (Fig. 3A and C). Because there were at least two types of *Runx2*-*neo* fusion transcripts in *Runx2-I^neo/neo^* mice (Fig. 3D and 3E, *middle panel*), it is possible that insertion of the *neo* cassette interfered with normal RNA splicing, as previously reported [15]–[18]. The impaired expression of Runx2 in the *Runx2-I^neo/neo^* mice (Fig. 5B, *left panel* *s*) led to significant impairment of bone-related-gene expression (Fig. 7A and B) and fetal loss of bone ossification (Figs 8–10), resulting in neonatal lethality of homozygous mice, as previously reported for *Runx2*-null mice [1], [2].

To our surprise, *neo*-depletion by crossing *Runx2^neo/+^* mice with Cre deleter mice resulted in the complete rescue of homozygous mice from lethality, even though these mice still had the mutation of the translation-initiating codon ATG to the stop codon TGA (*Runx2-I^TGA/TGA^* mice). By quantitative real-time PCR, we confirmed the restored expression of *Runx2* (Fig. 4D) and bone-related genes (Fig. 7C and D) in calvarial cells obtained from *Runx2-I^TGA/TGA^* mice; this result implies the existence of a sufficient amount of fully functional Runx2 protein in *Runx2-I^TGA/TGA^* mice. The next ATG is located on the first exon of *Runx2-I* in frame as the codon of the 25th amino acid of Runx2-I, and this ATG may have initiated the production of a truncated form of Runx2-I. Our data on the forced expression of the Runx2 isoforms demonstrates that the electrophoretic mobility of the two Runx2 isoforms did not differ enough for separation into two bands when the isoforms were mixed, and N-terminal-truncated Runx2 could be produced from each Runx2-expression vector (Fig. 5A). In addition, it has been reported that the two major bands detected by western blotting of wild-type calvarial cells could be attributed to Runx2 (upper band) and truncated Runx2 (lower band) [12]. Therefore, we suggest that the upper band in the blot for the wild-type control shown in Fig. 5B corresponds to Runx2-II, which is known to be the major isoform expressed in the murine fetus during this embryonic period [12], and that the lower band corresponds to a truncated form of Runx2. In mice with the *Runx2-I*-TGA allele, Runx2-I protein should be produced in an N-terminal-truncated form, owing to the loss of the first ATG. This truncated form may have merged with the lower band and consequently increased the signal density of that band (Fig. 5B, *right panel* *s*). This observation, taken together with the nonlethal phenotype of *Runx2-I^TGA/TGA^* mice, suggests that the isoform-specific N-terminal amino acid sequence of Runx2-I does not have a unique function in osteogenesis. Consistently, several previous reports showed that ectopic expression of a transgene for each *Runx2* variant readily induces skeletal ossification [21], [22]. Our findings strongly indicate that the two Runx2 isoforms exert their function in a total dose-dependent manner rather than an isoform-specific manner, although their expression is controlled spatiotemporally by unique promoters. In other words, the role of each Runx2 isoform is distinctive under the control of a unique promoter *in vivo*.

Curiously, when we crossed *Runx2-I^neo/+^* mice with Col2a1-Cre transgenic mice, which express Cre specifically in chondrocytes, the survival of Cre-*Runx2-I^neo/neo^* mice was comparable to that of other littermates (*Runx2-I^+/+^* 17; *Runx2-I^neo/+^* 31; *Runx2-I^neo/neo^* 11, among Col2a1-Cre-possessing mice at 3 weeks of age). This result indicates that fetal inability to breathe, observed in several Runx2-deficient mouse strains, was partly due to defective chondrogenesis rather than osteogenesis. The amount of *Runx2-I* transcript in tissues other than bone (e.g., spleen) of Cre-*Runx2-I^neo/neo^* mice was still significantly lower than that of wild-type mice (data not shown). Because *Runx2-I*, but not *Runx2-II*, is expressed in nonskeletal tissues such as lung and brain [8], [23], Col2a1-Cre-possessing *Runx2-I^neo/neo^* mice are useful for investigating the role of Runx2 in nonskeletal tissues.

Although at the time of dissection, the expression of osteogenic genes *Alpl* and *Tnfsf11* was impaired in the calvarial cells of *Runx2-I^neo/neo^* mice, the reduction in the expression of other genes, such as *Bglap* and *Sp7*, was less pronounced (Fig. 7A and data not shown). This result suggests that partial impairment of these genes can result in serious bone malformation *in vivo*. Consistent with this result, clavicles, the development of which starts the earliest in skeletogenesis and continues for the longest duration [24], are among the bones that are the most susceptible to altered expression of osteogenic genes [18]. We assume that the fact that no significant reduction in the levels of the transcripts of *Bglap* and *Sp7* was detected was due to the early examination time point, because other investigators measured the amounts of the same gene transcripts after the birth of mutant mice and observed significant changes between the wild-type and the mutants [20], [25]. In fact, we observed significantly reduced expression of *Bglap* and *Sp7* in *Runx2-I^neo/neo^* mice compared with expression in littermate controls after 14-day culture under osteoblast differentiating conditions (Fig. 7B).

Upon morphological examination, *Runx2-I^neo/+^* mice showed a phenotype intermediate between that of the wild-type and homozygotes (Figs. 8 and 9A); however, the quantified parameters of the heterozygotes did not differ from those of the wild-type (Fig. 9B). This result indicates that Runx2-I plays a dominant role in intramembranous ossification. This interpretation is consistent with previous reports showing that *Runx2-II* and *Runx2-I* are differentially expressed in the murine sagittal suture and that *Runx2-I* is more broadly expressed than *Runx2-II* [11], [26]. In addition, it has been reported that *Runx2-II* is responsible more for endochondral ossification than for intramembranous ossification [9]. Another possible explanation is that endochondrally ossified bones depend on the level of total *Runx2* transcripts to a lesser extent than do intramembranously ossified bones. Actually, in *Runx2*-null mice, weak calcification was found at the tibias and ulnas, both of which are endochondrally ossified [2], [12]. These previous results, together with our current results, suggest that there is some compensatory machinery in endochondrally ossified long bones even in the absence of Runx2. Further careful examination of both possibilities is needed to distinguish the role of each Runx2 isoform.

Taking the previous research into consideration [20], [25], we suggest that defective bone formation in *Runx2-I^neo/neo^* mice was the result of impaired osteoblast development as shown by the results of *in vitro* assays (Fig. 10). Importantly, cells of heterozygote origin showed an intermediate phenotype in these assays, implying that the effects of *Runx2* were dose dependent *in vitro*, the phenotype reflecting an intermediate level of defects in calvaria formation in these mice.

In this study, we provide novel insights into the functions and properties of Runx2 in general and the Runx2-I isoform in particular. Our results suggest that Runx2-I plays an essential role in intramembranous ossification even when its specific N-terminal amino acids are removed. For clinical application to cleidocranial dysplasia, examination of the role of the P2 promoter, which has received less attention than the role of the P1 promoter, will be of great value.

## References

[1] Otto F, Thornell AP, Crompton T, Denzel A, Gilmour KC, et al (1997) Cbfa1, a candidate gene for cleidocranial dysplasia syndrome, is essential for osteoblast differentiation and bone development. Cell 89: 765–771. 9182764 10.1016/s0092-8674(00)80259-7

[2] Komori T, Yagi H, Nomura S, Yamaguchi A, Sasaki K, et al (1997) Targeted disruption of Cbfa1 results in a complete lack of bone formation owing to maturational arrest of osteoblasts. Cell 89: 755–764. 9182763 10.1016/s0092-8674(00)80258-5

[3] Mundlos S, Otto F, Mundlos C, Mulliken JB, Aylsworth AS, et al (1997) Mutations involving the transcription factor CBFA1 cause cleidocranial dysplasia. Cell 89: 773–779. 9182765 10.1016/s0092-8674(00)80260-3

[4] Blyth K, Cameron ER, Neil JC (2005) The RUNX genes: gain or loss of function in cancer. Nature Reviews Cancer 5: 376–387. 15864279 10.1038/nrc1607

[5] Lam K, Zhang DE (2012) RUNX1 and RUNX1-ETO: roles in hematopoiesis and leukemogenesis. Frontiers in bioscience: a journal and virtual library 17: 1120–1139. 10.2741/3977 PMC3433167 22201794

[6] Rudra D, Egawa T, Chong MMW, Treuting P, Littman DR, et al (2009) Runx-CBFβ complexes control expression of the transcription factor Foxp3 in regulatory T cells. Nature immunology 10: 1170–1177. 19767756 10.1038/ni.1795 PMC2764816

[7] Ono M, Yaguchi H, Ohkura N, Kitabayashi I, Nagamura Y, et al (2007) Foxp3 controls regulatory T-cell function by interacting with AML1/Runx1. Nature 446: 685–689. 17377532 10.1038/nature05673

[8] Banerjee C, Javed A, Choi JY, Green J, Rosen V, et al (2001) Differential regulation of the two principal Runx2/Cbfa1 n-terminal isoforms in response to bone morphogenetic protein-2 during development of the osteoblast phenotype. Endocrinology 142: 4026–4039. 11517182 10.1210/endo.142.9.8367

[9] Stock M, Otto F (2005) Control of RUNX2 isoform expression: the role of promoters and enhancers. Journal of cellular biochemistry 95: 506–517. 15838892 10.1002/jcb.20471

[10] Sudhakar S, Li Y, Katz MS, Elango N (2001) Translational regulation is a control point in RUNX2/Cbfa1 gene expression. Biochemical and biophysical research communications 289: 616–622. 11716520 10.1006/bbrc.2001.6033

[11] Park MH, Shin HI, Choi JY, Nam SH, Kim YJ, et al (2001) Differential expression patterns of Runx2 isoforms in cranial suture morphogenesis. Journal of Bone and Mineral Research 16: 885–892. 11341333 10.1359/jbmr.2001.16.5.885

[12] Zhang S, Xiao Z, Luo J, He N, Mahlios J, et al (2009) Dose-Dependent Effects of Runx2 on Bone Development. Journal of Bone and Mineral Research 24: 1889–1904. 19419310 10.1359/JBMR.090502 PMC2765932

[13] Ogata N, Chikazu D, Kubota N, Terauchi Y, Tobe K, et al (2000) Insulin receptor substrate-1 in osteoblast is indispensable for maintaining bone turnover. Journal of Clinical Investigation 105: 935–954. 10749573 10.1172/JCI9017 PMC377487

[14] Nishikawa K, Nakashima T, Takeda S, Isogai M, Hamada M, et al (2010) Maf promotes osteoblast differentiation in mice by mediating the age-related switch in mesenchymal cell differentiation. The Journal of clinical investigation 120: 3455. 20877012 10.1172/JCI42528 PMC2947225

[15] Carmeliet P, Ferreira V, Breier G, Pollefeyt S, Kieckens L, et al (1996) Abnormal blood vessel development and lethality in embryos lacking a single VEGF allele. Nature 380: 435–439. 8602241 10.1038/380435a0

[16] Nagy A, Moens C, Ivanyi E, Pawling J, Gertsenstein M, et al (1998) Dissecting the role of N-myc in development using a single targeting vector to generate a series of alleles. Current biology: CB 8: 661–664. 9635194 10.1016/s0960-9822(98)70254-4

[17] Meyers EN, Lewandoski M, Martin GR (1998) An Fgf8 mutant allelic series generated by Cre- and Flp-mediated recombination. Nature genetics 18: 136–141. 9462741 10.1038/ng0298-136

[18] Lou Y, Javed A, Hussain S, Colby J, Frederick D, et al (2009) A Runx2 threshold for the cleidocranial dysplasia phenotype. Human molecular genetics 18: 556–568. 19028669 10.1093/hmg/ddn383 PMC2638795

[19] Choi JY, Pratap J, Javed A, Zaidi SK, Xing L, et al (2001) Subnuclear targeting of Runx/Cbfa/AML factors is essential for tissue-specific differentiation during embryonic development. Proceedings of the National Academy of Sciences 98: 8650–8655. 10.1073/pnas.151236498 PMC37490 11438701

[20] Xiao ZS, Hjelmeland AB, Quarles L (2004) Selective deficiency of the “bone-related” Runx2-II unexpectedly preserves osteoblast-mediated skeletogenesis. Journal of Biological Chemistry 279: 20307–20313. 15007057 10.1074/jbc.M401109200

[21] Takeda S, Bonnamy JP, Owen MJ, Ducy P, Karsenty G (2001) Continuous expression of Cbfa1 in nonhypertrophic chondrocytes uncovers its ability to induce hypertrophic chondrocyte differentiation and partially rescues Cbfa1-deficient mice. Genes & development 15: 467–481. 11230154 10.1101/gad.845101 PMC312629

[22] Ueta C, Iwamoto M, Kanatani N, Yoshida C, Liu Y, et al (2001) Skeletal malformations caused by overexpression of Cbfa1 or its dominant negative form in chondrocytes. The Journal of cell biology 153: 87–100. 11285276 10.1083/jcb.153.1.87 PMC2185519

[23] Jeong JH, Jin JS, Kim HN, Kang SM, Liu JC, et al (2008) Expression of Runx2 transcription factor in non-skeletal tissues, sperm and brain. Journal of cellular physiology 217: 511–517. 18636555 10.1002/jcp.21524 PMC2612588

[24] Hall BK (2001) Development of the clavicles in birds and mammals. The Journal of experimental zoology 289: 153–161. 11170011 10.1002/1097-010x(20010215)289:3<153::aid-jez1>3.0.co;2-o

[25] Liu JC, Lengner CJ, Gaur T, Lou Y, Hussain S, et al (2011) Runx2 Protein Expression Utilizes the Runx2 P1 Promoter to Establish Osteoprogenitor Cell Number for Normal Bone Formation. Journal of Biological Chemistry 286: 30057–30070. 21676869 10.1074/jbc.M111.241505 PMC3191046

[26] Lee MH, Kim YJ, Yoon WJ, Kim JI, Kim BG, et al (2005) Dlx5 specifically regulates Runx2 type II expression by binding to homeodomain-response elements in the Runx2 distal promoter. The Journal of biological chemistry 280: 35579–35587. 16115867 10.1074/jbc.M502267200

